# Buckling Analysis of Functionally Graded Sandwich Plates under Both Mechanical and Thermal Loads

**DOI:** 10.3390/ma14237194

**Published:** 2021-11-25

**Authors:** Dongdong Li, He Zhu, Xiaojing Gong

**Affiliations:** 1 State Key Laboratory of Mechanics and Control of Mechanical Structures, Nanjing University of Aeronautics and Astronautics, No. 29 Yudao Street, Nanjing 210016, China; zhuhe0728@nuaa.edu.cn; 2 Institut Clément Ader (ICA), CNRS UMR 5312, University of Toulouse, UPS, 1 rue Lautréamont, 65000 Tarbes, France; xiaojing.gong@iut-tarbes.fr; 3 Key Laboratory of Fundamental Science for National Defense-Advanced Design Technology of Flight Vehicle, Nanjing University of Aeronautics and Astronautics, Nanjing 210016, China

**Keywords:** functionally graded material, sandwich plate, thermomechanical load, buckling

## Abstract

This paper presents an analytical solution for the thermomechanical buckling of functionally graded material (FGM) sandwich plates. The solution is obtained using a four-variable equivalent-single-layer (ESL) plate theory. Two types of sandwich plates are included: one with FGM facesheets and homogeneous core, and vice versa for the other. The governing equations are derived based on the principle of minimum total potential energy. For simply supported boundary conditions, these equations are solved via the Navier method. The results on critical buckling load and temperature increment of simply supported FGM sandwich plates are compared with the available solutions in the literature. Several results are presented considering various material and geometrical parameters as well as their effect on the thermomechanical buckling response of FGM sandwich plates. The relationship between the mechanical load and the temperature increment for uniform/linear temperature rise of FGM sandwich plates under combined mechanical and thermal loads is studied.

## 1. Introduction

Sandwich structures have many benefits such as their light weight and high bending stiffness and thus have been broadly applied in aircraft, aerospace, flexible electronics, and biomedical areas [1,2]. A conventional sandwich structure has two homogeneous facesheets and a homogeneous core in between. Recently, the introduction of nonhomogeneous materials, such as the functionally graded materials (FGMs), has made sandwich structures become even more attractive [3]. As the options for the facesheets and/or the core, FGMs help reduce the interlaminar stresses and thermal stresses, enhancing the mechanical and thermal performances of sandwich structures [4,5,6]. In the literature, two types of FGM sandwich structures are mainly included: for type-A, sandwich structures with FGM facesheets and a homogeneous core [7,8,9,10,11,12,13], and vice versa for type-B [12,13,14,15,16,17]. FGM sandwich structures combine the dual advantages of sandwich structures and FGMs, and thus offer unique potential in many fields of engineering, including planetary exploration landers, return capsules, submarines, and so on [18,19].

As one of the critical failure modes, buckling behavior of FGM sandwich structures has been studied by a number of researchers. However, most of them are restricted to FGM sandwich plates under mechanical load [20,21,22,23,24,25,26,27,28,29,30,31,32,33,34] or thermal load [35,36,37,38,39,40,41,42]. Different methods are used, such as zig-zag plate theory, equivalent-single-layer (ESL) theories, unified formulation, and the mesh-free method. Very few publications considering both mechanical and thermal load can be found. In practice, FGM sandwich plates are commonly exposed to mechanical and thermal loads at the same time, so it is essential to include both for accurate prediction of the buckling problem. Based on a high-order ESL plate theory, Yang et al. [43] employed the Galerkin-differential quadrature approach to analyze the buckling of type-A FGM sandwich plates under mechanical load and uniform temperature increment. Shen and collaborators [44,45] studied the buckling and postbuckling of type-A FGM sandwich plates under mechanical and thermal loads via a two-step perturbation technique. Based on a first-order ESL plate theory, Yaghoobi and Yaghoobi [46] investigated the thermomechanical buckling of type-A FGM sandwich plates resting on an elastic foundation. Tung [47] conducted a thermomechanical postbuckling analysis of FGM sandwich plates under mechanical load and uniform temperature rise, using a first-order ESL plate theory. References [43,44,45,46,47] are mainly focused on type-A FGM sandwich plates or uniform temperature rise.

Based on the literature review, it was found that the limited amount of available work is not sufficient to provide a comprehensive understanding of buckling performance of FGM sandwich plates under mechanical and thermal loads. Recently, a four-variable ESL plate theory was developed to address the thermomechanical bending of FGM sandwich plates, which shows good accuracy and efficiency [12]. This theory has not been extended to the analysis of the thermomechanical buckling behavior of FGM sandwich plates. Thus, the objective of this article is to use this four-variable ESL plate theory to investigate the thermomechanical buckling problem of simply supported FGM sandwich plates. In this study, both type-A and type-B FGM sandwich plates are included. Both uniform temperature rise, and graded temperature rise are considered. Power-law FGM is adopted herein. The material properties are assumed to be temperature-independent [48,49]. The governing equations are obtained from the principle of minimum total potential energy and solved via Navier Method. The present theory was verified by comparing calculated results with those from the existing literature. Numerical results were calculated considering the effects of volume fraction distribution and geometrical parameters on the thermomechanical buckling of FGM sandwich plates. In this framework, a linear relationship between the mechanical load and thermal load is presented.

## 2. Theoretical Formulation

Consider a rectangular FGM sandwich plate with constant thickness *h*, length *a*, and width *b*. The Cartesian coordinate system *xyz* is taken such that the *xy* plane (*z* = 0) coincides with the geometric mid-surface of the sandwich plate. In the present study, the sandwich plate is subjected to a system of uniform, in-plane, compressive loads at the side edges and a thermal load through the thickness.

Both type-A and type-B FGM sandwich plates are included. Since the composition distribution in FGMs is designable, three typical gradations are commonly used in the literature: power law, exponential law, and sigmoid law [49,50]. In the present study, the power law is adopted to describe the ceramic volume fraction.

### 2.1. Type-A Sandwich Plate: Sandwich Plates with FGM Facesheets and Homogeneous Core

**Figure 1:**
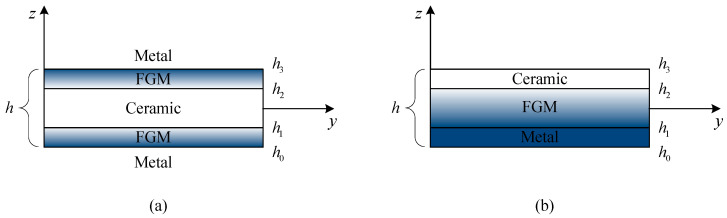
Two different types of FGM sandwich plates and in-plane loads: (**a**) type-A with FGM facesheets and homogeneous core; (**b**) type-B with homogeneous facesheets and FGM core.

In type-A sandwich plates, the sandwich core is homogeneous while the facesheets are functionally graded through the thickness, as shown in Figure 1. The ceramic volume fraction of type-A sandwich plate is given by

(1) $$\begin{array}{ll} V^{\left(1\right)}\;=\;{\left(\frac{z-h_{0}}{h_{1}-h_{0}}\right)}^{p} & z\in \left[h_{0},h_{1}\right]\\ V^{\left(2\right)}\;=\;1 & z\in \left[h_{1},h_{2}\right]\\ V^{\left(3\right)}\;=\;{\left(\frac{z-h_{3}}{h_{2}-h_{3}}\right)}^{p} & z\in \left[h_{2},h_{3}\right] \end{array}$$

where $V^{\left(n\right)}$ is the volume fraction of *n*-th layer and *p* is the power index greater than or equal to zero.

### 2.2. Type-B Sandwich Plate: Sandwich Plates with Homogeneous Facesheets and FGM Core

In type-B sandwich plates, the facesheets are homogeneous while the core layer is functionally graded through the thickness, as illustrated in Figure 1. The ceramic volume fraction of type-B sandwich plate is given as:

(2) $$\begin{array}{ll} V^{\left(1\right)}\;=\;0 & z\in \left[h_{0},h_{1}\right]\\ V^{\left(2\right)}\;=\;{\left(\frac{z-h_{1}}{h_{2}-h_{1}}\right)}^{p} & z\in \left[h_{1},h_{2}\right]\\ V^{\left(3\right)}\;=\;1 & z\in \left[h_{2},h_{3}\right] \end{array}$$

### 2.3. Material Properties

The effective material properties *P*^(*n*)^ for *n*-th layer, such as the Young’s modulus $E^{\left(n\right)}$ and the thermal expansion coefficient $\alpha^{\left(n\right)}$ at a point can be determined by the linear rule of mixture as

(3) $$P^{\left(n\right)}\left(z\right)\;=\;P_{m}\;+\;\left(P_{c}-P_{m}\right)V^{\left(n\right)}$$

where subscripts *m* and *c* represent metal and ceramic, respectively. Since the Poisson’s ratio ν varies only in a small range through the plate thickness, for simplicity, it is assumed to be constant [33,49].

### 2.4. Four-Variable ESL Plate Theory

An ESL plate theory containing four variables is used, of which the basic assumptions have been stated in references [51,52]. The displacement field of the present study is:

(4) $$\begin{array}{l} u\left(x,y,z\right)\;=\;u_{0}\left(x,y\right)\;-\;z\frac{\partial w_{b}}{\partial x}\;-\;f\left(z\right)\frac{\partial w_{s}}{\partial x}\\ v\left(x,y,z\right)\;=\;v_{0}\left(x,y\right)\;-\;z\frac{\partial w_{b}}{\partial y}\;-\;f\left(z\right)\frac{\partial w_{s}}{\partial y}\\ w\left(x,y,z\right)\;=\;w_{b}\left(x,y\right)\;+\;w_{s}\left(x,y\right) \end{array}$$

in which

(5) $$f\left(z\right)\;=\;\frac{4z^{3}}{3h^{2}}$$

where *u*, *v*, and *w* represent the displacements in the *x*, *y*, and *z* directions, respectively; $u_{0}$ and $v_{0}$ are the midplane displacements; $w_{b}$ and $w_{s}$ are the bending and shear parts of the transverse displacement *w*, respectively.

### 2.5. Geometric Equations

The non-linear von Karman strain–displacement equations are

(6) $$\begin{array}{l} \varepsilon_{x}\;=\;\frac{\partial u}{\partial x}\;+\;\frac{1}{2}{\left(\frac{\partial w}{\partial x}\right)}^{2}\\ \varepsilon_{y}\;=\;\frac{\partial v}{\partial y}\;+\;\frac{1}{2}{\left(\frac{\partial w}{\partial y}\right)}^{2}\\ \gamma_{xy}\;=\;\frac{\partial v}{\partial x}\;+\;\frac{\partial u}{\partial y}\;+\;\frac{\partial w}{\partial x}\frac{\partial w}{\partial y}\\ \gamma_{yz}\;=\;\frac{\partial w}{\partial y}\;+\;\frac{\partial v}{\partial z}\\ \gamma_{xz}\;=\;\frac{\partial w}{\partial x}\;+\;\frac{\partial u}{\partial z} \end{array}$$

Using Equations (4) and (6), we get

(7) $$\left\{\begin{array}{l} \varepsilon_{x}\\ \varepsilon_{y}\\ \gamma_{xy} \end{array}\right\}\;=\;\left\{\begin{array}{l} \varepsilon_{x}^{\left(0\right)}\\ \varepsilon_{y}^{\left(0\right)}\\ \gamma_{xy}^{\left(0\right)} \end{array}\right\}\;+\;z\left\{\begin{array}{l} k_{x}^{b}\\ k_{y}^{b}\\ k_{xy}^{b} \end{array}\right\}\;+\;f\left(z\right)\left\{\begin{array}{l} k_{x}^{s}\\ k_{y}^{s}\\ k_{xy}^{s} \end{array}\right\},\;\varepsilon_{z}\;=\;0,\;\left\{\begin{array}{l} \gamma_{yz}\\ \gamma_{xz} \end{array}\right\}\;=\;\left[1-f^{\prime }\left(z\right)\right]\left\{\begin{array}{l} \gamma_{yz}^{\left(0\right)}\\ \gamma_{xz}^{\left(0\right)} \end{array}\right\}$$

where

(8) $$\begin{array}{l} \left\{\begin{array}{l} \varepsilon_{x}^{\left(0\right)}\\ \varepsilon_{y}^{\left(0\right)}\\ \gamma_{xy}^{\left(0\right)} \end{array}\right\}\;=\;\left\{\begin{array}{c} \frac{\partial u_{0}}{\partial x}+\frac{1}{2}{\left(\frac{\partial w_{b}}{\partial x}+\frac{\partial w_{s}}{\partial x}\right)}^{2}\\ \frac{\partial v_{0}}{\partial y}+\frac{1}{2}{\left(\frac{\partial w_{b}}{\partial y}+\frac{\partial w_{s}}{\partial y}\right)}^{2}\\ \frac{\partial u_{0}}{\partial y}+\frac{\partial v_{0}}{\partial x}+\left(\frac{\partial w_{b}}{\partial x}+\frac{\partial w_{s}}{\partial x}\right)\left(\frac{\partial w_{b}}{\partial y}+\frac{\partial w_{s}}{\partial y}\right) \end{array}\right\},\;\\ \left\{\begin{array}{l} k_{x}^{b}\\ k_{y}^{b}\\ k_{xy}^{b} \end{array}\right\}\;=\;-\left\{\begin{array}{c} \frac{\partial^{2}w_{b}}{\partial x^{2}}\\ \frac{\partial^{2}w_{b}}{\partial y^{2}}\\ 2\frac{\partial^{2}w_{b}}{\partial x\partial y} \end{array}\right\},\;\left\{\begin{array}{l} k_{x}^{s}\\ k_{y}^{s}\\ k_{xy}^{s} \end{array}\right\}\;=\;-\left\{\begin{array}{c} \frac{\partial^{2}w_{s}}{\partial x^{2}}\\ \frac{\partial^{2}w_{s}}{\partial y^{2}}\\ 2\frac{\partial^{2}w_{s}}{\partial x\partial y} \end{array}\right\},\;\left\{\begin{array}{l} \gamma_{yz}^{\left(0\right)}\\ \gamma_{xz}^{\left(0\right)} \end{array}\right\}\;=\;\left\{\begin{array}{l} \frac{\partial w_{s}}{\partial y}\\ \frac{\partial w_{s}}{\partial x} \end{array}\right\} \end{array}$$

The stress–strain relationships accounting for thermal effects for the *n*-th layer can be written as

(9) $${\left\{\begin{array}{l} \sigma_{x}\\ \sigma_{y}\\ \tau_{yz}\\ \tau_{xz}\\ \tau_{xy} \end{array}\right\}}^{\left(n\right)}\;=\;{\left\{\begin{array}{l} \sigma_{x}^{0}\\ \sigma_{y}^{0}\\ \tau_{yz}^{0}\\ \tau_{xz}^{0}\\ \tau_{xy}^{0} \end{array}\right\}}^{\left(n\right)}\;+\;{\left\{\begin{array}{l} \sigma_{x}^{T}\\ \sigma_{y}^{T}\\ \tau_{yz}^{T}\\ \tau_{xz}^{T}\\ \tau_{xy}^{T} \end{array}\right\}}^{\left(n\right)}\;=\;{\left[\begin{array}{lllll} c_{11} & c_{12} & 0 & 0 & 0\\ c_{12} & c_{22} & 0 & 0 & 0\\ 0 & 0 & c_{44} & 0 & 0\\ 0 & 0 & 0 & c_{55} & 0\\ 0 & 0 & 0 & 0 & c_{66} \end{array}\right]}^{\left(n\right)}\left({\left\{\begin{array}{c} \varepsilon_{x}\\ \varepsilon_{y}\\ \gamma_{yz}\\ \gamma_{xz}\\ \gamma_{xy} \end{array}\right\}}^{\left(n\right)}-{\left\{\begin{array}{c} \alpha \Delta T\\ \alpha \Delta T\\ 0\\ 0\\ 0 \end{array}\right\}}^{\left(n\right)}\right),\;\left(n\;=\;1,\;2,\;3\right)$$

where Δ*T* is the temperature change from the stress-free state. The elastic constants $c_{ij}^{\left(n\right)}$ of the *n*-th layer are

(10) $$c_{11}^{\left(n\right)}\;=\;c_{22}^{\left(n\right)}\;=\;\frac{E^{\left(n\right)}\left(z\right)}{1-\nu^{2}},\;c_{12}^{\left(n\right)}\;=\;\nu c_{11}^{\left(n\right)},\;c_{44}^{\left(n\right)}\;=\;c_{55}^{\left(n\right)}\;=\;c_{66}^{\left(n\right)}\;=\;\frac{E^{\left(n\right)}\left(z\right)}{2\left(1+\nu \right)}$$

### 2.6. Governing Equations

Energy methods can commonly be taken to derive the governing equations, such as the total potential energy principle [53] and the principle of virtual displacements [54]. The total strain energy of the FGM sandwich plate can be written as

(11) $$U\;=\;\frac{1}{2}\int_{V}\left[\sigma_{x}^{0}\varepsilon_{x}+\sigma_{y}^{0}\varepsilon_{y}+\tau_{xy}^{0}\gamma_{xy}+\tau_{yz}^{0}\gamma_{yz}+\tau_{xz}^{0}\gamma_{yz}\right]dV\;+\;\frac{1}{2}\int_{V}\left[\sigma_{x}^{T}{\left(\frac{\partial w}{\partial x}\right)}^{2}+\sigma_{y}^{T}{\left(\frac{\partial w}{\partial y}\right)}^{2}\right]dV$$

The potential energy of external force is calculated by

(12) $$U_{e}\;=\;\frac{1}{2}\int_{\Omega }\left[N_{x}^{0}{\left(\frac{\partial w}{\partial x}\right)}^{2}+N_{y}^{0}{\left(\frac{\partial w}{\partial y}\right)}^{2}\right]d\Omega$$

where $N_{x}^{0}$ and $N_{y}^{0}$ represent distributed, compressive, in-plane forces in the *x* and *y* directions (per unit length).

In this study, the principle of minimum total potential energy is used, which takes the following form as

(13) $$\delta \left(U+U_{e}\right)\;=\;0$$

Substituting Equations (11) and (12) into Equation (13) obtains the governing equations of stability,

(14) $$\begin{array}{l} \frac{\partial N_{x}}{\partial x}\;+\;\frac{\partial N_{xy}}{\partial y}\;=\;0\\ \frac{\partial N_{xy}}{\partial x}\;+\;\frac{\partial N_{y}}{\partial y}\;=\;0\\ \frac{\partial^{2}M_{x}^{b}}{\partial x^{2}}\;+\;2\frac{\partial^{2}M_{xy}^{b}}{\partial x\partial y}\;+\;\frac{\partial^{2}M_{y}^{b}}{\partial y^{2}}\;+\;\bar{N}\;+\;{\bar{N}}^{T}\;=\;0\\ \frac{\partial^{2}M_{x}^{s}}{\partial x^{2}}\;+\;2\frac{\partial^{2}M_{xy}^{s}}{\partial x\partial y}\;+\;\frac{\partial^{2}M_{y}^{s}}{\partial y^{2}}\;+\;\frac{\partial Q_{xz}^{s}}{\partial x}\;+\;\frac{\partial Q_{yz}^{s}}{\partial y}\;+\;\bar{N}\;+\;{\bar{N}}^{T}\;=\;0 \end{array}$$

where the stress and moment resultants can be found in reference [12], and

(15) $$\begin{array}{l} \bar{N}\;=\;N_{x}^{0}\frac{\partial^{2}\left(w_{b}+w_{s}\right)}{\partial x^{2}}\;+\;N_{y}^{0}\frac{\partial^{2}\left(w_{b}+w_{s}\right)}{\partial y^{2}}\\ {\bar{N}}^{T}\;=\;-N_{x}^{T}\frac{\partial^{2}\left(w_{b}+w_{s}\right)}{\partial x^{2}}\;-\;N_{y}^{T}\frac{\partial^{2}\left(w_{b}+w_{s}\right)}{\partial y^{2}} \end{array}$$

in which

(16) $$\left\{\begin{array}{l} N_{x}^{T}\\ N_{y}^{T} \end{array}\right\}\;=\;\sum_{n=1}^{3}\int_{h_{n-1}}^{h_{n}}{\left\{\begin{array}{l} \left(c_{11}+c_{12}\right)\alpha T\\ \left(c_{12}+c_{22}\right)\alpha T \end{array}\right\}}^{\left(n\right)}dz$$

## 3. Solution Procedure for Eigenvalue Problems

For a simply supported FGM sandwich plate of which the boundary conditions are expressed as

(17) $$\begin{array}{l} x\;=\;0\;,\;a:v_{0}\;=\;w_{b}\;=\;w_{s}\;=\;0,\;\frac{\partial w_{s}}{\partial y}\;=\;0,\;N_{x}\;=\;0,\;M_{x}^{b}\;=\;M_{x}^{s}\;=\;0\\ y\;=\;0,\;b:u_{0}\;=\;w_{b}\;=\;w_{s}\;=\;0,\;\frac{\partial w_{s}}{\partial x}\;=\;0,\;N_{y}\;=\;0,\;M_{y}^{b}\;=\;M_{y}^{s}\;=\;0 \end{array}$$

close-form solutions can be found, because, using Navier procedure, the displacement field can be expanded as the following form:

(18) $$\left\{\begin{array}{l} u_{0}\\ v_{0}\\ w_{b}\\ w_{s} \end{array}\right\}\;=\;\sum_{m=1}^{\infty }\sum_{n=1}^{\infty }\left\{\begin{array}{c} U_{mn}\cos\left(\lambda x\right)\sin\left(\mu y\right)\\ V_{mn}\sin\left(\lambda x\right)\cos\left(\mu y\right)\\ W_{bmn}\sin\left(\lambda x\right)\sin\left(\mu y\right)\\ W_{smn}\sin\left(\lambda x\right)\sin\left(\mu y\right) \end{array}\right\}$$

where *U_mn_*, $V_{mn}$, $W_{bmn}$, and $W_{smn}$ are unknowns to be determined. λ = mπ/a and μ = nπ/b.

The critical buckling loads and temperature increment of FGM sandwich plates subjected to a system of uniform in-plane compressive loads $N_{x}^{0}$ and $N_{y}^{0}$ ($N_{xy}^{0}\;=\;0$) in thermal environment can be derived.

Assuming that there is a given ratio between $N_{x}^{0}$ and $N_{y}^{0}$ such that $N_{x}^{0}\;=\;-N_{0}$ and $N_{y}^{0}\;=\;-\gamma N_{0}$, by substituting Equation (18) into Equation (14), one can obtain

(19) $$\left(\left[K\right]-\left[\tilde{N}\right]-\left[{\tilde{N}}^{T}\right]\right)\left\{\Delta \right\}\;=\;0$$

where

(20) $$\left\{\Delta \right\}\;=\;{\left\{U_{mn}\;V_{mn}\;W_{bmn}\;W_{smn}\right\}}^{T}$$

The elements of matrix [*K*] can be found in reference [12]. Detailed expressions of matrices [$\tilde{N}$] and [${\tilde{N}}^{T}$] are listed in Appendix A.

For nontrivial solutions of Equation (19), the determinant $\det\left(\left[K\right]-\left[\tilde{N}\right]-\left[{\tilde{N}}^{T}\right]\right)$ should be equal to zero, which is written by

(21) $$\left|\left[K\right]-\left[\tilde{N}\right]-\left[{\tilde{N}}^{T}\right]\right|\;=\;0$$

Solving Equation (21) gives the critical buckling load with temperature increment or critical buckling temperature increment with in-plane compressive load, which will be presented in the following.

### 3.1. Critical Buckling Load

The critical buckling load without temperature increment is

(22) $$N_{0}^{cr}\;=\;\frac{\prod^{3}\left[\left(A_{11}D_{11}-B_{11}^{2}\right)H_{11}-C_{11}^{2}D_{11}-A_{11}F_{11}^{2}+2B_{11}C_{11}F_{11}\right]+a^{2}b^{2}\prod^{2}\left(A_{11}D_{11}-B_{11}^{2}\right)J_{44}}{\pi^{2}\left(\gamma a^{2}+b^{2}\right)a^{2}b^{2}\left\{\prod \left[\left(H_{11}+D_{11}-2F_{11}\right)A_{11}-{\left(C_{11}-B_{11}\right)}^{2}\right]+a^{2}b^{2}A_{11}J_{44}\right\}}$$

where *A*_11_, *B*_11_, *C*_11_, *D*_11_, *F*_11_, *H*_11_, *J*_44_ can be found in reference [12]:

(23) $$\prod \;=\;\left(a^{2}+b^{2}\right)\pi^{2}$$

The critical buckling load with temperature increment is

(24) $$N_{0}^{\prime }\;=\;N_{0}^{cr}\;-\;\frac{a^{2}b^{2}\left(\lambda^{2}N_{x}^{T}+\mu^{2}N_{y}^{T}\right)}{\pi^{2}\left(\gamma a^{2}+b^{2}\right)}$$

### 3.2. Critical Buckling Temperature Increment under Uniform Temperature Rise

In this case, the temperature of the FGM sandwich plate is uniformly raised from initial temperature $T_{i}$ to final temperature $T_{f}$ in which the sandwich plate buckles. The temperature increment is $\Delta T\;=\;T_{f}\;-\;T_{i}$.

By solving Equation (21), the critical buckling temperature increment without in-plane compressive load is shown to be

(25) $$\Delta T_{cr}\;=\;\frac{\prod^{2}\left[\left(A_{11}D_{11}-B_{11}^{2}\right)H_{11}-C_{11}^{2}D_{11}-A_{11}F_{11}^{2}+2B_{11}C_{11}F_{11}\right]+a^{2}b^{2}\prod \left(A_{11}D_{11}-B_{11}^{2}\right)J_{44}}{a^{2}b^{2}\beta_{1}\left\{\prod \left[\left(H_{11}+D_{11}-2F_{11}\right)A_{11}-{\left(C_{11}-B_{11}\right)}^{2}\right]+a^{2}b^{2}A_{11}J_{44}\right\}}$$

where

(26) $$\beta_{1}\;=\;\sum_{n=1}^{3}\int_{h_{n-1}}^{h_{n}}\frac{\alpha^{\left(n\right)}\left(z\right)E^{\left(n\right)}\left(z\right)}{1-\nu }dz$$

The critical buckling temperature increment with in-plane compressive load is

(27) $$\Delta T^{\prime }\;=\;\Delta T_{cr}\;-\;\frac{a^{2}b^{2}N_{0}\left(\lambda^{2}+\gamma \mu^{2}\right)}{\prod \beta_{1}}$$

### 3.3. Critical Buckling Temperature Increment under Graded Temperature Rise through the Plate Thickness

The top surface temperature $T_{t}$ is different from the bottom surface temperature $T_{b}$, which varies through the plate thickness according to

(28) $$T\left(z\right)\;=\;\Delta T{\left(\frac{z}{h}+\frac{1}{2}\right)}^{\xi }\;+\;T_{t}$$

In which $\Delta T\;=\;T_{t}\;-\;T_{b}$ is the buckling temperature difference and ξ is the temperature index (0 < ξ < ∞).

The critical buckling temperature increment without in-plane compressive load is

(29) $$\Delta T_{cr}\;=\;\frac{\prod^{2}\left[\left(A_{11}D_{11}-B_{11}^{2}\right)H_{11}-C_{11}^{2}D_{11}-A_{11}F_{11}^{2}+2B_{11}C_{11}F_{11}\right]+a^{2}b^{2}\prod \left(A_{11}D_{11}-B_{11}^{2}\right)J_{44}}{a^{2}b^{2}\beta_{2}\left\{\prod \left[\left(H_{11}+D_{11}-2F_{11}\right)A_{11}-{\left(C_{11}-B_{11}\right)}^{2}\right]+a^{2}b^{2}A_{11}J_{44}\right\}}\;-\;\frac{T_{t}\beta_{1}}{\beta_{2}}$$

where

(30) $$\beta_{2}\;=\;\sum_{n=1}^{3}\int_{h_{n-1}}^{h_{n}}\frac{\alpha^{\left(n\right)}\left(z\right)E^{\left(n\right)}\left(z\right)}{1-\nu }{\left(\frac{z}{h}+\frac{1}{2}\right)}^{\xi }dz$$

The critical buckling temperature increment with in-plane compressive load is

(31) $$\Delta T^{\prime }\;=\;\Delta T_{cr}\;-\;\frac{a^{2}b^{2}N_{0}\left(\lambda^{2}+\gamma \mu^{2}\right)}{\prod \beta_{2}}$$

## 4. Numerical Results

In this section, comparative study is given to verify the current formulation. Considering the influence of volume fraction distribution and geometric parameters, as well as mechanical and thermal loads, several results are presented to provide sufficient insight to the thermomechanical buckling of FGM sandwich plates. Typical material properties for metal and ceramic used in the numerical examples are listed in Table 1.

**Figure 2:**
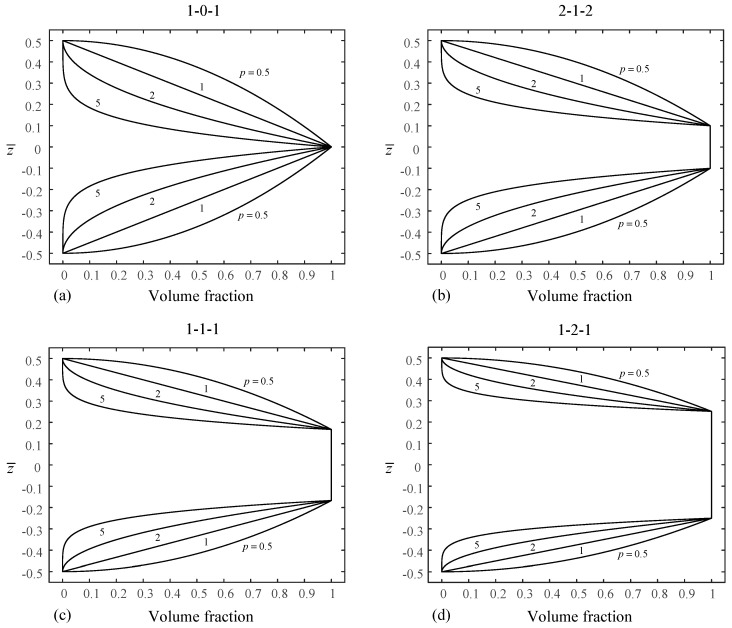
Variation of volume fraction through the plate thickness of four different kinds of type-A sandwich plates: (**a**) (1-0-1) sandwich plate, (**b**) (2-1-2) sandwich plate, (**c**) (1-1-1) sandwich plate, and (**d**) (1-2-1) sandwich plate.

**Figure 3:**
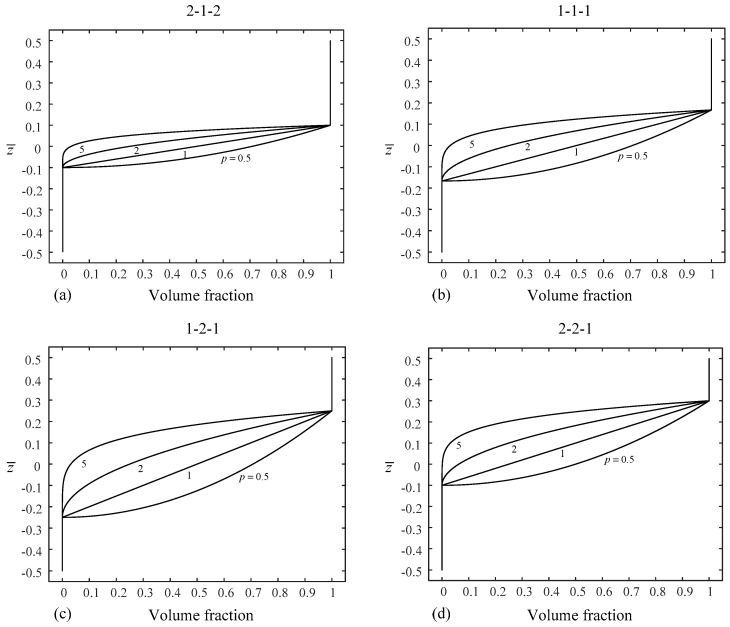
Variation of volume fraction through the plate thickness of four different kinds of type-B sandwich plates: (**a**) (2-1-2) sandwich plate, (**b**) (1-1-1) sandwich plate, (**c**) (1-2-1) sandwich plate, and (**d**) (2-2-1) sandwich plate.

For convenience of expression and illustration, the sandwich plates are denoted according to the layer thickness ratio of the facesheets and core. For example, (1-2-1) sandwich plate has a total thickness of *h*, core thickness of *h*/2, and two facesheets of equal thickness of *h*/4, and hence, in Figure 1, $h_{1}\;=\;-h/4$, $h_{2}\;=\;h/4$. Figure 2 and Figure 3 show the through-the-thickness variation of the ceramic volume fraction in type-A and type-B FGM sandwich plates for various values of *p* = {0.5, 1, 2, 5}. Unless otherwise specified, *a/h* = 10, $T_{t}$ = 25 K and γ = 1.

### 4.1. Validation Study

The validation study is conducted from the following four aspects: mechanical buckling of type-A and type-B FGM sandwich plates, as well as thermal buckling of type-A and type-B FGM sandwich plates. For mechanical buckling, the FGM sandwich plates are made from aluminum (Al) and alumina (Al_2_O_3_), while for thermal buckling, they are made from titanium (Ti-6Al-4V) and zirconia (ZrO_2_).

For better comparison and illustration, the following relations are adopted [11,35]:

(32) $$\begin{array}{l} \bar{z}\;=\;\frac{z}{h}\\ {\bar{N}}_{0}\;=\;\frac{N_{0}^{cr}a^{2}}{100h^{3}E_{0}}\\ T_{cr}\;=\;10^{-3}\Delta T_{cr} \end{array}$$

where $E_{0}$ = 1 GPa.

Table 2, Table 3, Table 4 and Table 5 shows the critical buckling load ${\bar{N}}_{0}$ and temperature increment of type-A and type-B sandwich plates for various layer thickness ratio and power index *p*. Results calculated by other theories in the literature are also presented as benchmark results. As observed, for every aspect of the buckling problem, an excellent agreement is reached.

### 4.2. Buckling Analysis of Type-A Sandwich Plate under Mechanical and Thermal Loads

In this example, a simply supported, square, type-A sandwich plate under the effect of mechanical and thermal loads is considered. The combination of materials consists of titanium and zirconia. It is assumed that the plate is subjected to mechanical load $N_{0}^{\prime }$ and temperature increment $\Delta T^{\prime }$ under uniform or linear temperature rise in which the plate buckles.

Dimensionless mechanical load and temperature rise are used as

(33) $$\begin{array}{l} {\hat{N}}_{0}\;=\;\frac{N_{0}^{\prime }a^{2}}{100h^{3}E_{0}}\\ \Delta \hat{T}\;=\;\frac{10^{-3}\Delta T^{\prime }}{T_{0}} \end{array}$$

where $T_{0}$ = 1 K.

Firstly, we calculate the dimensionless critical buckling load and temperature increment under uniform/linear temperature rise for type-A square sandwich plates for *p* = 0.5 and 2, and various layer thickness ratios of 1-0-1, 2-1-2, 1-1-1, and 1-2-1. Then we apply 1/2 of the critical buckling temperature increment under uniform temperature rise to calculate the mechanical load. In addition, we apply 1/2 of the critical buckling load to calculate the temperature increment under uniform/linear temperature rise. These results are given in Table 6. ${\hat{N}}_{T/2}$ represents the dimensionless mechanical load with 1/2 of the critical buckling temperature increment under uniform temperature rise; $T_{cr}^{u}$ and $T_{cr}^{l}$ represent the dimensionless critical temperature increment under uniform and linear temperature rise, respectively; ${\hat{T}}_{N/2}^{u}$ and ${\hat{T}}_{N/2}^{l}$ denote the dimensionless temperature increment under uniform and linear temperature rise with 1/2 of the critical buckling load. As observed, ${\hat{N}}_{T/2}\;=\;\frac{1}{2}{\bar{N}}_{0}$ and ${\hat{T}}_{N/2}^{u}\;=\;\frac{1}{2}T_{cr}^{u}$, but ${\hat{T}}_{N/2}^{l}\;<\;\frac{1}{2}T_{cr}^{l}$.

The relationship between ${\hat{N}}_{0}$ and $\Delta \hat{T}$ can be obtained from Equations (22), (24), (25), (27), (29) and (31).

For the uniform rise in temperature case,

(34) $$\frac{{\hat{N}}_{0}}{{\bar{N}}_{0}}\;+\;\frac{\Delta \hat{T}}{T_{cr}}\;=\;1$$

For the graded rise in temperature case,

(35) $$\frac{{\hat{N}}_{0}}{{\bar{N}}_{0}}\;+\;\frac{\Delta \hat{T}+\frac{10^{-3}T_{t}\beta_{1}}{T_{0}\beta_{2}}}{T_{cr}+\frac{10^{-3}T_{t}\beta_{1}}{T_{0}\beta_{2}}}\;=\;1$$

**Figure 4:**
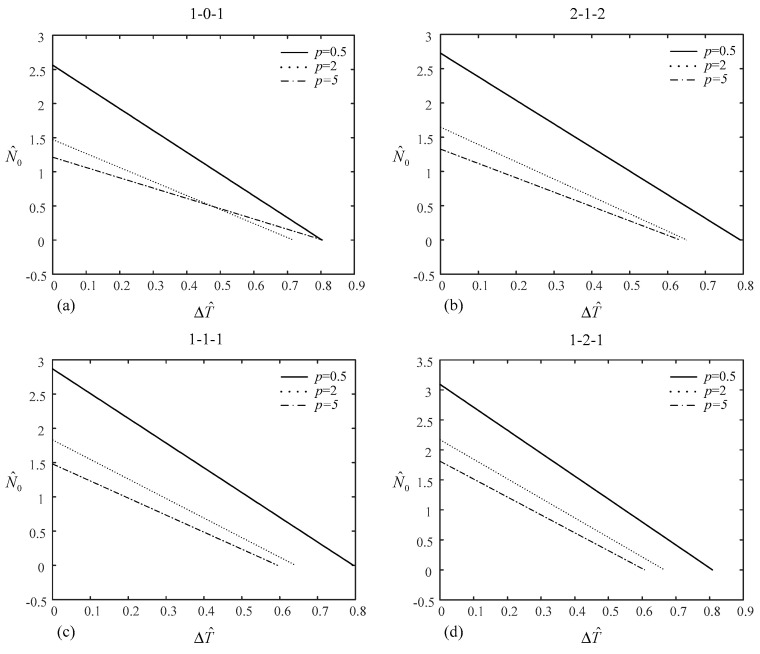
Relationship between the temperature increment under uniform temperature rise and the mechanical load of type-A sandwich plate: (**a**) (1-0-1) sandwich plate, (**b**) (2-1-2) sandwich plate, (**c**) (1-1-1) sandwich plate, (**d**) (1-2-1) sandwich plate.

**Figure 5:**
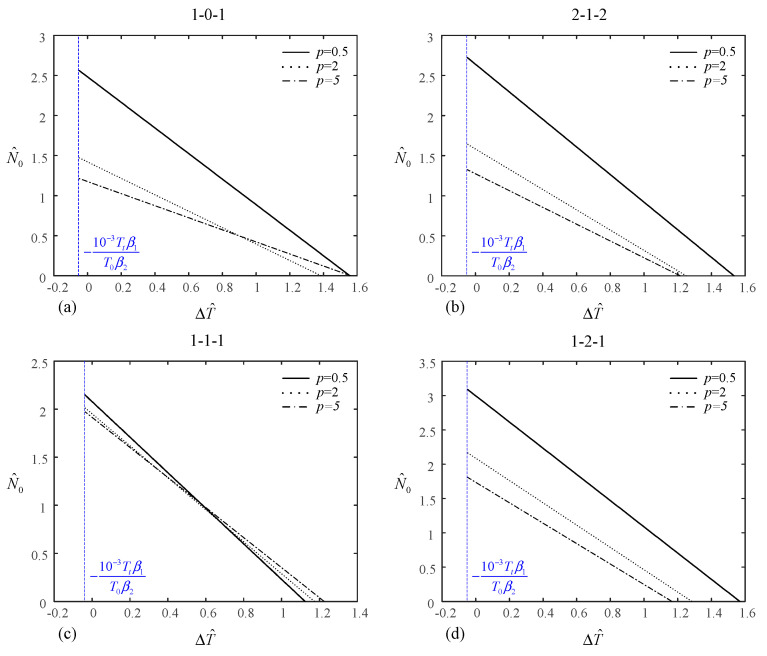
Relationship between the temperature increment under linear temperature rise and the mechanical load of type-A sandwich plate: (**a**) (1-0-1) sandwich plate, (**b**) (2-1-2) sandwich plate, (**c**) (1-1-1) sandwich plate, (**d**) (1-2-1) sandwich plate.

Figure 4 and Figure 5 depict the relationship between temperature increment under uniform/linear temperature rise and mechanical load of the (1-0-1), (2-1-2), (1-1-1), and (1-2-1) type-A sandwich plates for *p* = 0.5, 2, and 5. The relationship is linear, which can also be seen from Equations (34) and (35). With the increase in the temperature increment, the mechanical load decreases. This is expected, because a rise in temperature results in compressive internal force. In Figure 4, when the value of temperature increment reaches its maximum, the value of mechanical load is zero and vice versa. However, in Figure 5, when the value of mechanical load reaches its maximum, the value of temperature increment is $-\frac{10^{-3}T_{t}\beta_{1}}{T_{0}\beta_{2}}$. This is the reason why ${\hat{T}}_{N/2}^{l}\;<\;\frac{1}{2}T_{cr}^{l}$ in Table 6.

### 4.3. Buckling Analysis of Type-B Sandwich Plate under Mechanical and Thermal Loads

Similar work was also carried out for type-B FGM (Ti-6Al-4V/ZrO_2_) sandwich plates under the effect of mechanical and thermal loads.

**Figure 6:**
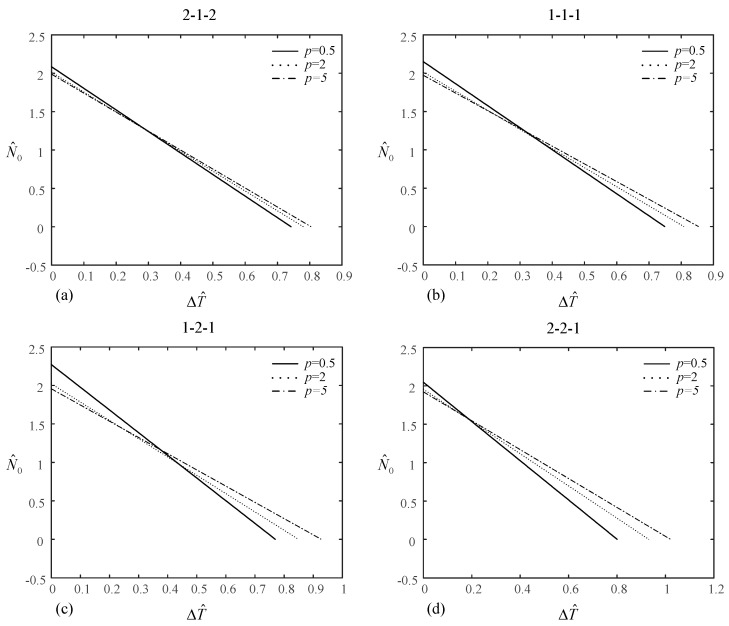
Relationship between the temperature increment under uniform temperature rise and the mechanical load of type-B sandwich plate: (**a**) (2-1-2) sandwich plate, (**b**) (1-1-1) sandwich plate, (**c**) (1-2-1) sandwich plate, (**d**) (2-2-1) sandwich plate.

**Figure 7:**
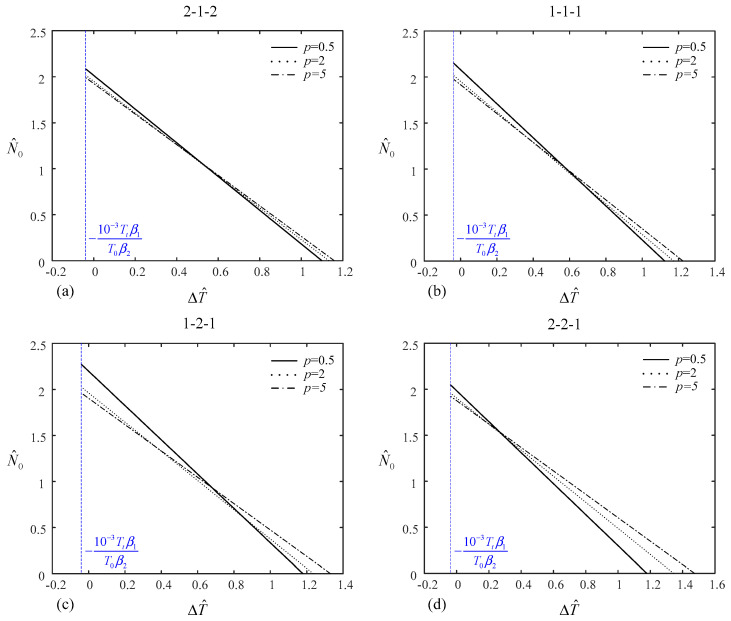
Relationship between the temperature increment under linear temperature rise and the mechanical load of type-B sandwich plate: (**a**) (2-1-2) sandwich plate, (**b**) (1-1-1) sandwich plate, (**c**) (1-2-1) sandwich plate, (**d**) (2-2-1) sandwich plate.

Table 7 shows the values of dimensionless mechanical load with 1/2 of the critical buckling temperature increment under uniform temperature rise and dimensionless temperature increment under uniform and linear temperature rise with 1/2 of the critical buckling load for various layer thickness ratios and power index *p*. Figure 6 and Figure 7 plot the relationship between temperature increment under uniform/linear temperature rise and mechanical load of the (2-1-2), (1-1-1), (1-2-1), and (2-2-1) type-B sandwich plates for *p* = 0.5, 2, and 5. Similar conclusions can be drawn.

## 5. Conclusions

Buckling analysis of FGM sandwich plates under thermomechanical load was performed using a four-variable ESL plate theory. Two different types of FGM sandwich plates were included: for type-A, sandwich plates with FGM facesheets and homogeneous core, and vice versa for type-B. The governing equations were deduced based on the principle of minimum total potential energy. The analytical solutions for simply supported boundary conditions were obtained using the Navier method. Critical buckling load and temperature increment under uniform, linear, and nonlinear temperature rise were calculated and compared with those published in the literature to demonstrate the accuracy of the present theory. Numerical studies were conducted considering the influences of volume fraction distribution and geometrical parameters on the thermomechanical buckling behavior of FGM sandwich plates. Some highlighted and interesting findings were obtained as follows:

(1) For both type-A and type-B FGM sandwich plates, the proposed formulation was found to be accurate. The present investigation extends the application range of this four-variable ESL plate theory.
(2) The critical buckling load with temperature increment and the critical buckling temperature increment with in-plane compressive load were presented. Both uniform temperature rise and graded temperature rise were taken into account.
(3) A linear relationship between the mechanical load and the temperature increment in which an FGM sandwich plate buckles was established. This relationship appears to be compact.

## Figures and Tables

**Table 1:** Material properties used in the FGM sandwich plates.

	Al	Ti-6Al-4V	ZrO_2_	Al_2_O_3_
Young’s modulus (GPa)	70	66.2	244.27	380
Poisson’s ratio	0.3	0.3	0.3	0.3
Coefficient of thermal expansion (10^−6^/K)	23	10.3	12.766	-

**Table 2:** Dimensionless critical buckling load ${\bar{N}}_{0}$ of type-A square sandwich plate (*a/h* = 10, *γ* = 1).

*p*	Theory	${\bar{N}}_{0}$					
		1-0-1	2-1-2	2-1-1	1-1-1	2-2-1	1-2-1
0	Reference [8]	6.5030	6.5030	6.5030	6.5030	6.5030	6.5030
	Reference [22] *ε_z_* ≠ 0	6.4764	6.4764	6.4764	6.4764	6.4764	6.4764
	Reference [22] *ε_z_* = 0	6.5025	6.5025	6.5025	6.5025	6.5025	6.5025
	Reference [28]	6.4774	6.4774	6.4774	6.4774	6.4774	6.4774
	Present	6.5025	6.5025	6.5025	6.5025	6.5025	6.5025
1	Reference [8]	2.5842	2.9206	3.0973	3.2327	3.4749	3.7531
	Reference [22] *ε_z_* ≠ 0	2.5307	2.8557	3.0273	3.1575	3.3920	3.6600
	Reference [22] *ε_z_* = 0	2.5392	2.8651	3.0368	3.1678	3.4027	3.6718
	Reference [28]	2.6190	2.9603	3.1401	3.2701	3.5129	3.7799
	Present	2.5836	2.9200	3.0970	3.2324	3.4747	3.7533
5	Reference [8]	1.3300	1.5220	1.7022	1.7903	2.0564	2.3674
	Reference [22] *ε_z_* ≠ 0	1.3183	1.5040	1.6813	1.7650	2.0254	2.3235
	Reference [22] *ε_z_* = 0	1.3234	1.5093	1.6860	1.7707	2.0308	2.3303
	Reference [28]	1.3552	1.5754	1.7636	1.8511	2.1227	2.4192
	Present	1.3291	1.5213	1.7018	1.7898	2.0560	2.3673
10	Reference [8]	1.2448	1.3742	1.5672	1.5973	1.5729	2.1909
	Reference [22] *ε_z_* ≠ 0	1.2361	1.3602	1.5303	1.5788	1.8308	2.1028
	Reference [22] *ε_z_* = 0	1.2411	1.3654	1.5347	1.5842	1.8358	2.1090
	Reference [28]	1.2553	1.4200	1.5995	1.6531	1.9133	2.1827
	Present	1.2436	1.3732	1.5459	1.5974	1.8538	2.1400

**Table 3:** Dimensionless critical buckling load ${\bar{N}}_{0}$ of type-B square sandwich plate (*γ* = 1).

*a/h*	Scheme	Theory	*p*				
			0	0.5	1	5	10
5	1-1-1	Reference [25]	2.0513	2.2342	2.3333	2.5978	2.6834
		Present	2.8714	2.5362	2.3782	2.1198	2.0769
	1-2-1	Reference [25]	1.9456	2.2725	2.4387	2.8964	3.0545
		Present	3.3388	2.7432	2.4697	2.0546	1.9894
	2-2-1	Reference [25]	2.1369	2.5023	2.7056	3.2351	3.4009
		Present	2.5905	2.3186	2.1891	1.9531	1.9031
10	1-1-1	Reference [25]	2.3508	2.5165	2.6123	2.8848	2.9773
		Present	3.1396	2.7889	2.6288	2.3970	2.3696
	1-2-1	Reference [25]	2.3095	2.5768	2.7322	3.2063	3.3816
		Present	3.6812	3.0330	2.7447	2.3697	2.3353
	2-2-1	Reference [25]	2.3928	2.7898	3.0116	3.6028	3.7937
		Present	2.8278	2.5746	2.4660	2.3189	2.2967
100	1-1-1	Reference [25]	2.4773	2.6308	2.7236	2.9969	3.0918
		Present	3.2397	2.8840	2.7238	2.5054	2.4857
	1-2-1	Reference [25]	2.4730	2.7015	2.8495	3.3268	3.5087
		Present	3.8104	3.1429	2.8497	2.4965	2.4781
	2-2-1	Reference [25]	2.4963	2.9038	3.1320	3.7467	3.9476
		Present	2.9161	2.6723	2.5738	2.4723	2.4657

**Table 4:** Critical buckling temperature increments *T_cr_* of type-A square sandwich plate under uniform temperature rise.

Scheme	*p*	Theory	*a/h*				
			5	10	15	25	50
1-0-1	0.5	Reference [35]	2.87276	0.80328	0.36504	0.13294	0.03340
		Present	2.87074	0.80314	0.36501	0.13294	0.03340
	2	Reference [35]	2.63459	0.71815	0.32462	0.11789	0.02958
		Present	2.63018	0.71783	0.32456	0.11788	0.02958
2-1-2	0.5	Reference [35]	2.83194	0.79232	0.36010	0.13116	0.03295
		Present	2.83030	0.79220	0.36008	0.13115	0.03295
	2	Reference [35]	2.39953	0.65098	0.29396	0.10671	0.02677
		Present	2.39637	0.65075	0.29392	0.10670	0.02677
1-1-1	0.5	Reference [35]	2.83331	0.79463	0.36134	0.13164	0.03308
		Present	2.83224	0.79456	0.36133	0.13164	0.03308
	2	Reference [35]	2.36195	0.64253	0.29031	0.10541	0.02645
		Present	2.36000	0.64239	0.29029	0.10541	0.02645
1-2-1	0.5	Reference [35]	2.86992	0.80925	0.36841	0.13430	0.03376
		Present	2.86972	0.80925	0.36841	0.13430	0.03376
	2	Reference [35]	2.42899	0.66689	0.30189	0.10972	0.02754
		Present	2.42873	0.66687	0.30189	0.10972	0.02754

**Table 5:** Critical buckling temperature increments *T_cr_* of type-B square sandwich plate under nonlinear temperature rise.

Scheme	*p*	*ξ*	Theory	*a/h*				
				5	10	15	25	50
1-0-1	0.5	2	Reference [42]	5.35784	1.46297	0.63750	0.20009	0.01238
			Present	5.36625	1.46432	0.63784	0.20010	0.01235
		3	Reference [42]	6.77704	1.85048	0.80636	0.25309	0.01565
			Present	6.78766	1.85219	0.80679	0.25311	0.01562
		4	Reference [42]	8.25929	2.25522	0.98272	0.30845	0.01908
			Present	8.27222	2.25729	0.98325	0.30846	0.01904
		5	Reference [42]	9.78867	2.67282	1.16470	0.36557	0.02261
			Present	9.80398	2.67527	1.16531	0.36558	0.02257
	2	2	Reference [42]	5.35784	1.46297	0.63750	0.20009	0.01238
			Present	5.36625	1.46432	0.63784	0.20010	0.01235
		3	Reference [42]	6.77704	1.85048	0.80636	0.25309	0.01565
			Present	6.78766	1.85219	0.80679	0.25311	0.01562
		4	Reference [42]	8.25929	2.25522	0.98272	0.30845	0.01908
			Present	8.27222	2.25729	0.98325	0.30846	0.01904
		5	Reference [42]	9.78867	2.67282	1.16470	0.36557	0.02261
			Present	9.80398	2.67527	1.16531	0.36558	0.02257
2-1-2	0.5	2	Reference [42]	5.45505	1.48364	0.64572	0.20226	0.01205
			Present	5.46373	1.48503	0.64607	0.20227	0.01203
		3	Reference [42]	6.94175	1.88799	0.82171	0.25738	0.01534
			Present	6.95278	1.88976	0.82216	0.25739	0.01531
		4	Reference [42]	8.48806	2.30855	1.00475	0.31471	0.01875
			Present	8.50153	2.31070	1.00529	0.31473	0.01871
		5	Reference [42]	10.07658	2.74059	1.19278	0.37361	0.02226
			Present	10.09256	2.74314	1.19343	0.37363	0.02222
	2	2	Reference [42]	5.43958	1.49762	0.65488	0.20728	0.01496
			Present	5.44823	1.49904	0.65525	0.20729	0.01493
		3	Reference [42]	6.81619	1.87662	0.82061	0.25973	0.01875
			Present	6.82701	1.87840	0.82107	0.25975	0.01871
		4	Reference [42]	8.24945	2.27122	0.99316	0.31435	0.02269
			Present	8.26253	2.27338	0.99372	0.31437	0.02265
		5	Reference [42]	9.72967	2.67876	1.17137	0.37075	0.02676
			Present	9.74508	2.68129	1.17203	0.37078	0.02671
1-1-1	0.5	2	Reference [42]	5.61588	1.52788	0.66526	0.20871	0.01289
			Present	5.62505	1.52936	0.66565	0.20873	0.01286
		3	Reference [42]	7.17052	1.95084	0.84943	0.26648	0.01645
			Present	7.18222	1.95273	0.84992	0.26651	0.01643
		4	Reference [42]	8.77713	2.38794	1.03974	0.32619	0.02014
			Present	8.79143	2.39025	1.04034	0.32622	0.02011
		5	Reference [42]	10.41987	2.83488	1.23434	0.38724	0.02391
			Present	10.43684	2.83761	1.23506	0.38728	0.02387
	2	2	Reference [42]	5.55788	1.54041	0.67549	0.21523	0.01728
			Present	5.56691	1.54193	0.67590	0.21525	0.01726
		3	Reference [42]	6.92118	1.91826	0.84118	0.26802	0.02152
			Present	6.93240	1.92015	0.84168	0.26805	0.02149
		4	Reference [42]	8.33175	2.30921	1.01262	0.32265	0.02591
			Present	8.34523	2.31148	1.01322	0.32268	0.02587
		5	Reference [42]	9.78507	2.71201	1.18925	0.37893	0.03043
			Present	9.80089	2.71467	1.18996	0.37897	0.03039
1-2-1	0.5	2	Reference [42]	5.93289	1.62019	0.70680	0.22290	0.01524
			Present	5.94305	1.62187	0.70726	0.22293	0.01522
		3	Reference [42]	7.60147	2.07586	0.90558	0.28558	0.01953
			Present	7.61446	2.07800	0.90616	0.28563	0.01951
		4	Reference [42]	9.30782	2.54184	1.10886	0.34969	0.02392
			Present	9.32371	2.54446	1.10957	0.34975	0.02388
		5	Reference [42]	11.04050	3.01502	1.31528	0.41479	0.02837
			Present	11.05936	3.01812	1.31612	0.41486	0.02833
	2	2	Reference [42]	5.77776	1.61506	0.71083	0.22846	0.02076
			Present	5.78748	1.61675	0.71129	0.22850	0.02074
		3	Reference [42]	7.14462	1.99715	0.87899	0.28251	0.02567
			Present	7.15663	1.99922	0.87956	0.28256	0.02564
		4	Reference [42]	8.54124	2.38754	1.05081	0.33773	0.03069
			Present	8.55556	2.39002	1.05149	0.33779	0.03065
		5	Reference [42]	9.96988	2.78688	1.22657	0.39423	0.03582
			Present	9.98657	2.78978	1.22737	0.39429	0.03578

**Table 6:** Dimensionless mechanical load and temperature change of type-A square sandwich plate.

Scheme	*p*	${\bar{N}}_{0}$	${\hat{N}}_{T/2}$	$T_{cr}^{u}$	${\hat{T}}_{N/2}^{u}$	$T_{cr}^{l}$	${\hat{T}}_{N/2}^{l}$
1-0-1	0.5	2.56201	1.28101	0.80314	0.40157	1.55628	0.75314
	2	1.47204	0.73602	0.71783	0.35892	1.38567	0.66783
2-1-2	0.5	2.72753	1.36376	0.79220	0.39610	1.53441	0.74220
	2	1.64738	0.82369	0.65075	0.32538	1.25150	0.60075
1-1-1	0.5	2.86964	1.43482	0.79456	0.39728	1.53912	0.74456
	2	1.83213	0.91606	0.64239	0.32119	1.23478	0.59239
1-2-1	0.5	3.09327	1.54664	0.80925	0.40463	1.56850	0.75925
	2	2.16916	1.08458	0.66687	0.33344	1.28375	0.61687

**Table 7:** Dimensionless mechanical load and temperature change of type-B square sandwich plate.

Scheme	*p*	${\bar{N}}_{0}$	${\hat{N}}_{T/2}$	$T_{cr}^{u}$	${\hat{T}}_{N/2}^{u}$	$T_{cr}^{l}$	${\hat{T}}_{N/2}^{l}$
2-1-2	0.5	2.08427	1.04213	0.74185	0.37093	1.09927	0.53046
	2	2.01363	1.00682	0.77997	0.38998	1.13338	0.54793
1-1-1	0.5	2.15063	1.07532	0.74858	0.37429	1.12358	0.54238
	2	2.01452	1.00726	0.80801	0.40401	1.17291	0.56773
1-2-1	0.5	2.27147	1.13574	0.76941	0.38471	1.17961	0.57000
	2	2.02209	1.01104	0.84871	0.42435	1.23624	0.59936
2-2-1	0.5	2.04786	1.02393	0.80101	0.40051	1.17799	0.57002
	2	1.95690	0.97845	0.93147	0.46574	1.34424	0.65359

## Data Availability

Not applicable.

## Appendix A

(A1) $$\left[\tilde{N}\right]\;=\;\left[\begin{array}{cccc} 0 & 0 & 0 & 0\\ 0 & 0 & 0 & 0\\ 0 & 0 & k & k\\ 0 & 0 & k & k \end{array}\right],\;\left[{\tilde{N}}^{T}\right]\;=\;\left[\begin{array}{cccc} 0 & 0 & 0 & 0\\ 0 & 0 & 0 & 0\\ 0 & 0 & l & l\\ 0 & 0 & l & l \end{array}\right]$$

in which

(A2) $$\begin{array}{l} k\;=\;N_{0}\left(\lambda^{2}+\gamma \mu^{2}\right)\\ l\;=\;\lambda^{2}N_{x}^{T}\;+\;\mu^{2}N_{y}^{T} \end{array}$$
